# Loss of PKM2 in Lgr5^+^ intestinal stem cells promotes colitis-associated colorectal cancer

**DOI:** 10.1038/s41598-019-42707-8

**Published:** 2019-04-17

**Authors:** Yeji Kim, Yong-Soo Lee, Sung Wan Kang, Seungil Kim, Tae-Young Kim, Su-Hyun Lee, Sung Wook Hwang, Jihun Kim, Eun Na Kim, Jin-Sung Ju, Yun-Yong Park, Mi-Na Kweon

**Affiliations:** 1 Mucosal Immunology Laboratory, Department of Convergence Medicine, University of Ulsan College of Medicine/Asan Medical Center, Seoul, Republic of Korea; 2 Department of Gastroenterology, University of Ulsan College of Medicine/Asan Medical Center, Seoul, Republic of Korea; 3 Department of Pathology, University of Ulsan College of Medicine/Asan Medical Center, Seoul, Republic of Korea; 4 Department of Convergence Medicine, University of Ulsan College of Medicine/Asan Medical Center, Seoul, Republic of Korea

**Keywords:** Cancer metabolism, Colon cancer

## Abstract

The regulatory properties of pyruvate kinase M2 isoform (PKM2), the key glycolytic enzyme, influence altered energy metabolism including glycolysis in cancer. In this study, we found that PKM2 was highly expressed in patients with ulcerative colitis or colorectal cancer (CRC). We then investigated the effectiveness of conditionally ablating PKM2 in Lgr5^+^ intestinal stem cells (ISC) using a mouse model of colitis-associated CRC (AOM plus DSS). Tamoxifen-inducible Lgr5-driven deletion of PKM2 in ISC (PKM2^ΔLgr5^-Tx) significantly promoted tumor incidence and size in the colon and lower body weight compared with findings in vehicle-treated mice (PKM2^ΔLgr5^-Veh). Histopathologic analysis revealed considerable high-grade dysplasia and adenocarcinoma in the colon of PKM2^ΔLgr5^-Tx mice while PKM2^ΔLgr5^-Veh mice had low- and high-grade dysplasia. Loss of PKM2 was associated with dominant expression of PKM1 in Lgr5^+^ ISC and their progeny cells. Further, the organoid-forming efficiency of whole cancer cells or Lgr5^+^ cells obtained from colon polyps of PKM2^ΔLgr5^-Tx mice was significantly increased when compared with PKM2^ΔLgr5^-Veh mice. Cancer organoids from PKM2^ΔLgr5^-Tx mice exhibited increased mitochondrial oxygen consumption and a shift of metabolites involved in energy metabolism. These findings suggest that loss of PKM2 function in ISC promotes colitis-associated CRC.

## Introduction

Pyruvate kinase (PK) is involved in the final step of glycolysis by transferring the phosphate group from phosphoenolpyruvate to ADP and producing pyruvate and ATP. It has four isozymes (L, R, M1, and M2), which exhibit different kinetic properties at the expressing tissues^1^. Among them, the M2 isoform of PK (PKM2) has received the most attention due to its involvement in the Warburg effect in cancer. Most cancer cells utilize aerobic glycolysis, known as the Warburg effect, to facilitate the synthesis of cellular building blocks (amino acids, nucleotides, and lipids) needed to produce a new cell^2^. Previous studies suggested that PKM2 is a key glycolytic enzyme that is involved in aerobic glycolysis and anabolic metabolism in cancer cells^3,4^. Lower activity of PKM2 in tumor cells, which exists in a dimeric form, could allow the accumulation of glycolytic intermediates to enter the glycolysis branch pathway^5^. In addition to its metabolic function, PKM2 can promote transcriptional activities through interactions with Oct-4^6^, HIF-1α^7^, STAT3^8^, and β-catenin^9^. These PKM2 functions might support metabolic reprogramming and progression of cancer.

Although the critical role of PKM2 in cancer development is well known, recent studies have yielded conflicting results about the requirement for PKM2. Loss of PKM2 in a mouse model of breast cancer resulted in accelerated tumor growth and mortality^10^. In another study, knockdown of PKM2 led to impairment of tumor cell proliferation *in vitro* and had no effect on *in vivo* tumor xenograft growth^11^. In addition, a recent study showed that depletion of PKM2 did not affect c-MYC-induced liver tumor formation^12^. These results challenge the idea that PKM2 is essential in tumorigenesis.

PKM2 is the dominant isoform in normal colon tissues and is overexpressed in tumor-induced colon tissues^13,14^. Also, PKM2 levels in serum and feces were elevated in inflammatory bowel disease (IBD) patients^15,16^. Patients with IBD, such as Crohn’s disease and ulcerative colitis (UC), are at high risk of developing colorectal cancer (CRC). More than 20% of patients with IBD develop colon cancer within 30 years^17^. Genetic mutations in cancer-related regulatory genes, caused by chronic inflammation and oxidative stress, result in abnormal growth of intestinal epithelial cells and the pathogenesis of colitis-associated CRC^18,19^. Despite its elevation in IBD and CRC, the exact role of PKM2 in pathogenesis remains to be determined.

Intestinal epithelium undergoes continuous cell renewal throughout adulthood^20^. The self-renewing of intestinal epithelium is mediated by intestinal stem cells (ISC), which exist at the base of intestinal crypts. Lgr5 (leucine-rich-repeat containing G-protein-coupled receptor 5), a marker for ISC^21^, is also expressed in tumor-induced colon tissues of both humans and mice^22^. Lgr5-specific-loss of *APC* (adenomatous polyposis coli) led to progressively growing intestinal adenoma, while *APC* deletion in other cell types did not form adenoma^23^. When Lgr5-GFP mice were treated with azoxymethane (AOM) and dextran sodium sulfate (DSS) to induce inflammation-driven CRC, 75% of the polyps arose from GFP^+^Lgr5^+^ sites^24^. Therefore, these results suggest that Lgr5 acts as a marker for cancer stem cells (CSC).

In this study, we examined the potential role of PKM2 on development of inflammation-induced CRC. We addressed PKM2 expression in patients with UC or CRC. Mice with specific deletion of PKM2 in Lgr5^+^ or Villin^+^ cells exhibited enhanced tumor progression in the AOM/DSS-induced CRC murine model. Loss of PKM2 in Lgr5^+^ or Villin^+^ cells resulted in enhancement of PKM1 in steady-state and CRC conditions. Our findings challenge the idea that PKM2 functions as an oncogene.

## Results

### PKM2 as an oncogenic factor in inflammation-induced CRC

**Figure 1:**
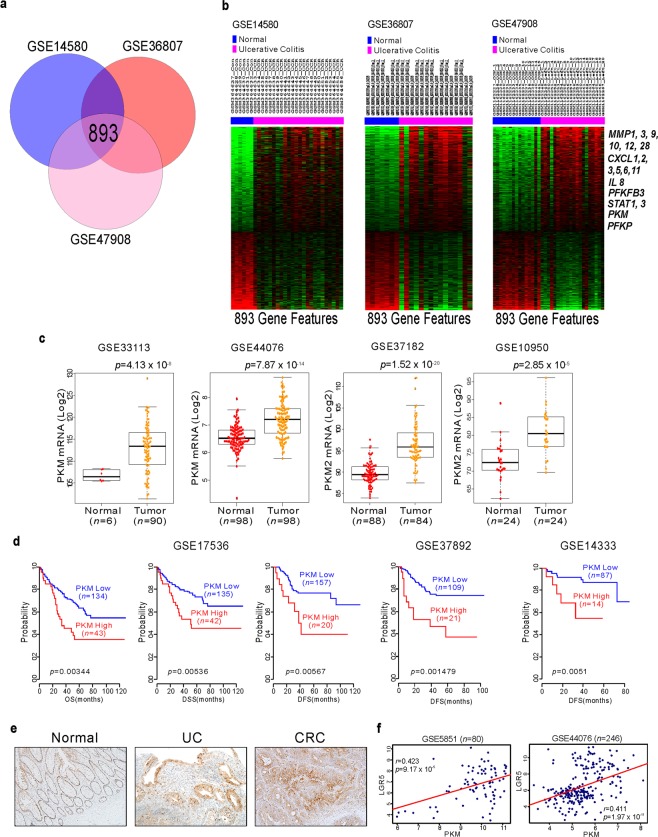
PKM2 expression and clinical implication in ulcerative colitis (UC) and colorectal cancer (CRC). (**a**) Venn diagram of genes showing significant differential expression between normal and UC tissue in three different patient cohorts. A univariate test (two-sample *t*-test) with a multivariate permutation test (10,000 random permutations) was employed. In each comparison, a cut-off *p*-value of <0.001 was applied to retain genes with an expression level that differed significantly between the two groups of tissues examined. Expression of 893 genes was commonly up- or down-regulated in all three cohorts. (**b**) Genes involved in inflammation and metabolism are highlighted in bold text. (**c**) PKM gene expression from multiple CRC patient cohorts. *P*-values show significance of expression between two groups. (**d**) Indicated CRC patient cohorts were dichotomized by relatively high or low PKM gene expression using the best cutoff-based on median values and then used for plotting. (**e**) Immunohistochemistry staining of PKM2 in normal (left), UC (middle), and CRC (right) patients. Original magnification, x200. (**f**) Scatter plots and correlation of Lgr5 and PKM gene expression from indicated CRC cohorts. Data represent mean ± s.d. of indicated samples. Student’s *t*-test was used to examine statistical significance.

Ulcerative colitis (UC) is a contributing factor to CRC. To elucidate which factors are involved in UC, we performed genomic analysis of previously reported gene expression data [GSE14580, GSE36807, and GSE47908; Gene Expression Omnibus (GEO) in the National Center for Biotechnology Information]^25–27^. To explore for genes differentially expressed in UC, we compared normal colon to UC samples by applying class comparison analysis. We found 893 genes that were potentially UC correlated (Fig. 1a). As expected, since UC is highly correlated with inflammation, genes (MMPs, CXCLs, STATs, and IL-8) associated with inflammation were significantly up-modulated in UC patients. Genes involved in glycolysis such as PFKFB3, PKM, and PFKP were highly increased in UC patients (Fig. 1b). Previous reports demonstrate that PFKFB3 is associated with UC and CRC^28^. However, there has been little characterization of the function of PKM in inflammation-induced CRC. We thus focused on PKM function in UC and CRC. First, we examined PKM expression in the colon tissues of CRC patients. As shown in Fig. 1c, PKM expression in those tissues was significantly higher than in normal tissue. We next investigated the clinical relevance of PKM and CRC. Patient cohorts from the GEO were dichotomized according to PKM expression. Patients with higher PKM levels had poor clinical outcomes and vice versa across multiple sample sets (Fig. 1d), consistent with an oncogenic function of PKM in CRC. Others have reported that PKM2 is the predominant isoform in normal colon epithelial cells and colon cancers^13,14^. We also found that mRNA levels of PKM2, but not PKM1, were highly expressed in both human normal and cancer colon tissues (data not shown). Thus, we can speculate that PKM from gene expression profile data represent PKM2 rather than PKM1. To determine whether PKM2 expression is changed during inflammation and colon oncogenesis, we used immunohistochemistry (IHC) to validate expression of PKM2 in UC and CRC. Indeed, PKM2 expression was increased in UC tissues and greater yet in dysplastic tumors (Fig. 1e). Moreover, in CRC patients, the expression of Lgr5, the cancer stem cell marker, was positively correlated with PKM expression (Fig. 1f). These results suggest that PKM2 expression is increased during inflammation-induced CRC development, either as a consequence of oncogenic transformation or as a metabolic oncogene.

### PKM2 deletion in Lgr5^+^ ISC or Villin^+^ epithelial cells aggravates inflammation-induced CRC

**Figure 2:**
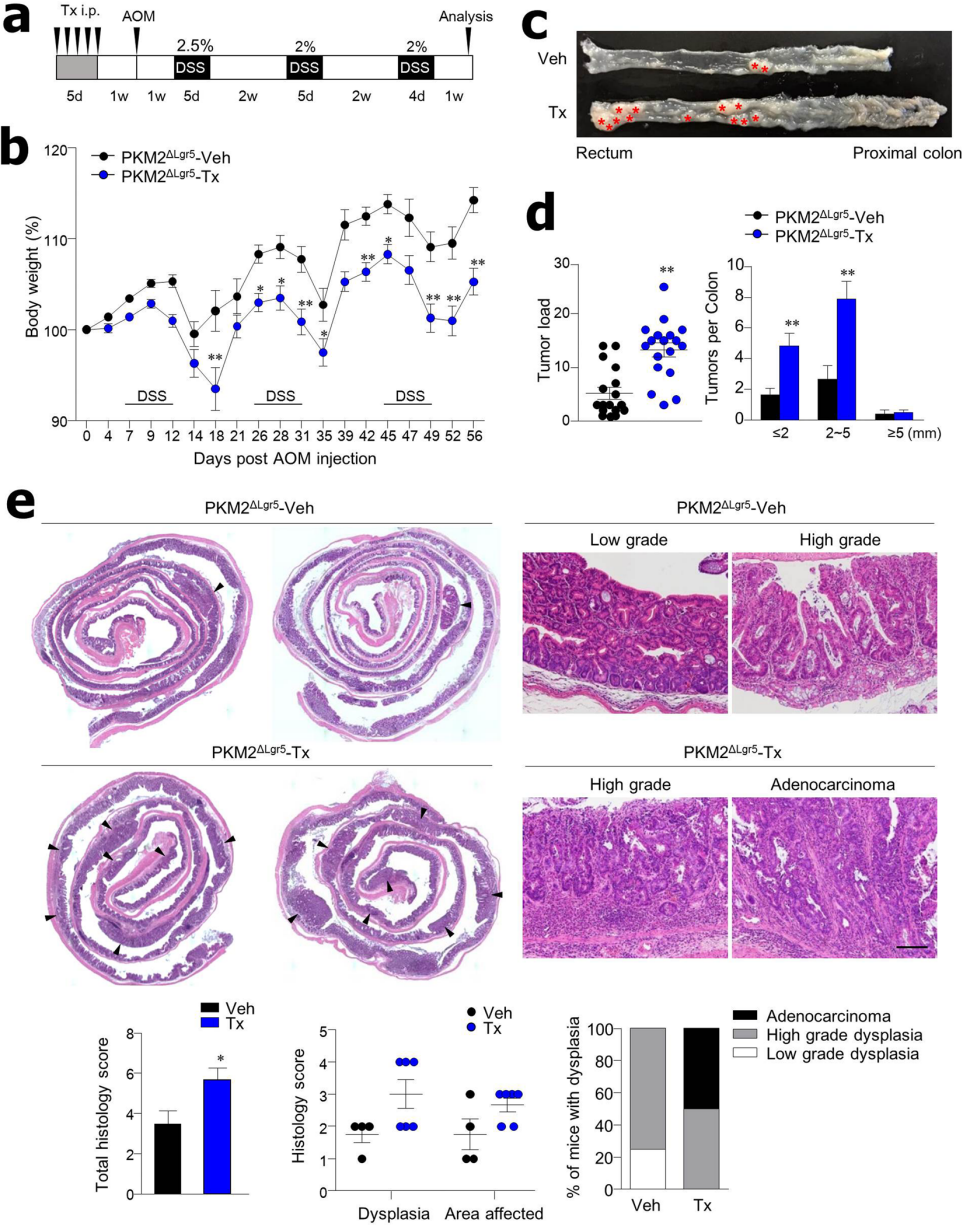
Lgr5^+^ specific-deletion of PKM2 accelerates development of inflammation-induced CRC. (**a**) Treatment scheme for AOM/DSS colon cancer model. PKM2^f/f^xLgr5^CreERT2^ mice were injected with vehicle (Veh) or tamoxifen (Tx) following induction of colon cancer by treatment of AOM and DSS. (**b**) Weight loss (n = 32) and (**c**) representative images of colons from AOM/DSS-treated PKM2^ΔLgr5^ mice. (**d**) Tumor load (n = 17 for Veh, n = 18 for Tx) and tumor size (n = 15 for Veh, n = 17 for Tx) in AOM/DSS-treated mice. (**e**) Colon histology of AOM/DSS-treated mice. Arrowheads indicate colon polyps. Representative images of low- and high-grade dysplasia and adenocarcinoma. Scale bar = 100 μm. H&E stained sections were scored for severity and area of dysplasia (n = 4 for Veh, n = 6 for Tx). All data are mean ± s.e.m. Statistical analyses were done by Student’s *t*-test or two-way ANOVA with Bonferroni *post-hoc* test. *p < 0.05, **p < 0.01.

To investigate the regulatory role of PKM2 in ISC, PKM2^f/f^ mice were crossed with Lgr5^CreERT2^ mice. We used tamoxifen treatment to show specific deletion of PKM2 in GFP-expressing Lgr5^+^ ISC and their progeny (Fig. S1) and a combination of AOM and DSS treatment to induce inflammation-derived CRC (Fig. 2a). PKM2^f/f^xLgr5^CreERT2^-Tamoxifen (PKM2^ΔLgr5^-Tx) mice had more significant body weight loss (Fig. 2b) and increased morbidity (87.23% vs. 71.43%, data not shown) when compared to PKM2^f/f^xLgr5^CreERT2^-Vehicle (PKM2^ΔLgr5^-Veh) mice. The tumor load was significantly higher in the colon of PKM2^ΔLgr5^-Tx mice than in PKM2^ΔLgr5^-Veh mice while tumor size distribution was comparable in PKM2^ΔLgr5^-Veh and -Tx mice (Fig. 2c,d). About half of the PKM2^ΔLgr5^-Tx mice developed adenocarcinoma while PKM2^ΔLgr5^-Veh mice had low- and high-grade dysplasia. Tumors progressed to more severe dysplasia and areas affected by dysplasia were larger in the colons of PKM2^ΔLgr5^-Tx mice than in PKM2^ΔLgr5^-Veh mice (Fig. 2e). The acceleration of tumor growth associated with PKM2 loss was further confirmed in mice lacking PKM2 in intestinal epithelial cells including ISC (PKM2^ΔIEC^ mice), which were generated by crossbreeding with PKM2^f/f^ and Villin^Cre^ mice (Fig. S2A–D). These results suggest that the deletion of PKM2 in Lgr5^+^ ISC or whole epithelial cells accelerates inflammation-induced colon tumor growth.

### Cancer cells with PKM2 depletion express high levels of PKM1

**Figure 3:**
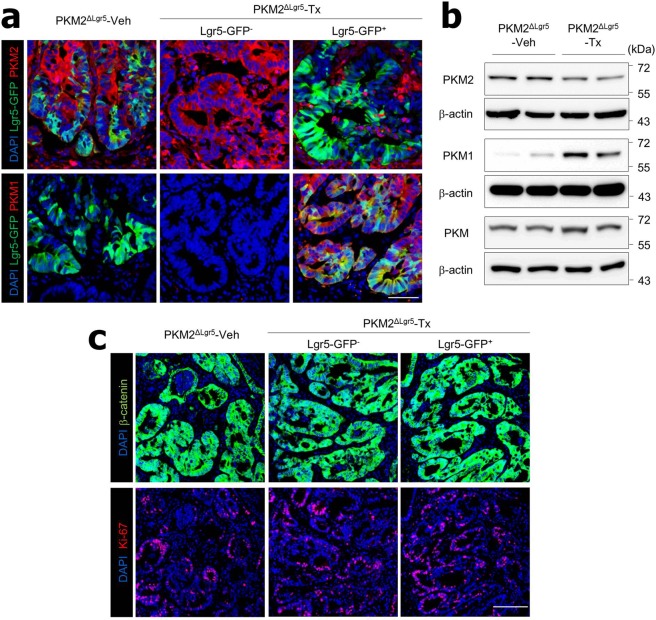
Cancer cells with PKM2 deletion express PKM1. (**a**) Immunofluorescence images of PKM2 and PKM1 expression and (**b**) Western blot analysis of colon polyp tissues from AOM/DSS-treated PKM2^ΔLgr5^–Veh and –Tx mice. The blots were cropped. Each target gene and the control gene were run on the same gel. The full-length blots are presented in Fig. S5B. (**c**) Confocal analysis of β-catenin and Ki67 expression in colon polyps from AOM/DSS-treated PKM2^ΔLgr5^ mice. Scale bar = 50 μm (**a**) and 100 μm (**c**). Data are representative of three independent experiments.

As reported by others^13,14^, we found high levels of PKM2 but not PKM1 expression in the colon epithelium of PKM2^f/f^ mice in the steady state (Fig. S3A,B). Interestingly, highly activated PKM1 but not PKM2 expression was found in the epithelium of PKM2^ΔIEC^ mice in the steady state (Fig. S3A,B). After tamoxifen treatment of PKM2^f/f^xLgr5^CreERT2^ mice, PKM2 expression was depleted in the epithelium of Lgr5-GFP^+^ cells but there were no changes in Lgr5-GFP^−^ cells (Fig. S1A). PKM2-depleted Lgr5-GFP^+^ cells expressed PKM1 in the steady-state condition (Fig. S1A). We assessed expression patterns of PKM1 and PKM2 in the colon polyps after treatment with AOM plus DSS. PKM2-intact Lgr5-GFP^−^ or Lgr5-GFP^+^ ISC-derived cancer cells in colon polyps did not express PKM1 in PKM2^ΔLgr5^-Tx or PKM2^ΔLgr5^-Veh mice (Fig. 3a). In contrast, PKM2-null cancer cells that originated from Lgr5-GFP^+^ cells in colon polyps of PKM2^ΔLgr5^-Tx mice expressed mainly PKM1 (Fig. 3a). Western blot analysis showed higher levels of PKM1 in normal colon epithelial cells (Fig. S1B) and polyp tissues (Fig. 3b) in PKM2^ΔLgr5^-Tx mice than in PKM2^ΔLgr5^-Veh mice. Total levels of PKM2 protein in normal colon epithelium (Fig. S1B) and polyp tissues (Fig. 3b) were slightly lower in PKM2^ΔLgr5^-Tx mice than in PKM2^ΔLgr5^-Veh mice as PKM2 was partially depleted in the intestines (i.e., Lgr5^+^ cells only). The levels of total PKM, including PKM1 and PKM2, were identical in the two groups (Figs 3b and S1B). Because PKM2 is involved in epidermal growth factor receptor-promoted β-catenin transactivation^9^ and PKM1 causes proliferation arrest^29^, we next assessed β-catenin activation and cell proliferation. We found no significant differences in expression of nuclear translocation of β-catenin or numbers of Ki67-positive proliferating cells in PKM2- and PKM1-positive cells in colon polyp tissues (Fig. 3c). These results indicate that cancer cells originating from PKM2-depleted Lgr5^+^ ISC activate PKM1 expression and depletion of PKM2 does not attenuate β-catenin activation and cell proliferation.

### Loss of PKM2 in Lgr5^+^ ISC increases cancer stem cell-like function

**Figure 4:**
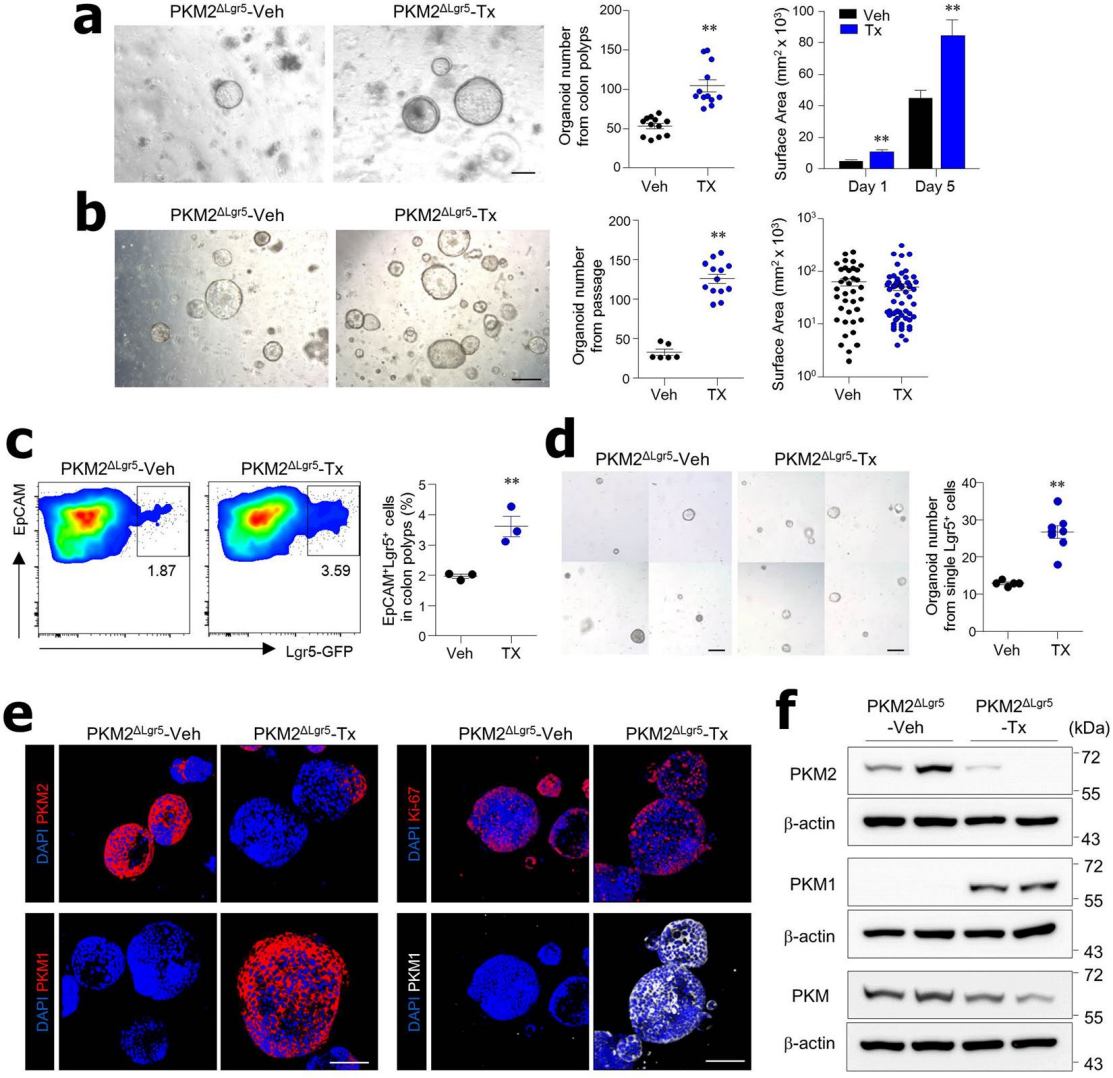
Formation and growth of organoids from colon polyps of PKM2^ΔLgr5^ mice. (**a**) Bright field images and quantification (number and size) of primary organoids from colon polyps of AOM/DSS-treated PKM2^ΔLgr5^–Veh and –Tx mice. Surface area was measured at ≥50 organoids. Scale bar = 200 μm. (**b**) Bright field images and quantification (number and size) of secondary passaged organoids. Organoids were seeded at 5 × 10^3^ cells/well. Surface area was measured at ≥30 organoids. Scale bar = 500 μm. (**c**) Quantification by flow cytometry analysis of EpCAM^+^ Lgr5-GFP^+^ cells in colon polyps of AOM/DSS-treated PKM2^ΔLgr5^–Veh and –Tx mice. (**d**) Bright field images and numbers of formed organoids from sorted single EpCAM^+^ Lgr5-GFP^+^ cells isolated from colon polyps of AOM-DSS treated PKM2^ΔLgr5^-Veh and PKM2^ΔLgr5^-Tx mice. Sorted cells were cultured in medium without R-spondin and Wnt3a. Scale bar = 200 μm. (**e**) Immunofluorescence staining of PKM1, PKM2, and Ki-67 in cancer organoids isolated from colon polyps of PKM2^ΔLgr5^–Veh and –Tx mice. Scale bar = 100 μm. (**f**) Western blot analysis of cancer organoids from colon polyps of PKM2^ΔLgr5^–Veh and –Tx mice. The full-length blots are presented in Fig. S6. The Data are representative of two independent experiments. All data are mean ± s.e.m. Statistical analyses were done by Student’s *t*-test. **p < 0.01.

To further examine the effects of PKM2 loss on tumor growth, we isolated cells from colon polyps of AOM/DSS-treated PKM2^ΔLgr5^ mice and cultured them in matrigel for organoid formation. To ensure cancer organoid formation, isolated cells from colon polyps were cultured in medium without R-spondin and Wnt3a, which are essential for normal organoids^30^. PKM2^ΔLgr5^-Tx mice had larger organoids and were significantly more efficient in forming cancer organoids than the PKM2^ΔLgr5^-Veh mice (Fig. 4a), indicating that PKM2^ΔLgr5^-Tx mice had more adenoma mutations within the Wnt pathway. When cancer organoids were dissociated to single cells, those from PKM2^ΔLgr5^-Tx mice still maintained their elevated ability for organoid formation when compared with the PKM2^ΔLgr5^-Veh mice (Fig. 4b). The fact that surface area per organoid did not differ between PKM2^ΔLgr5^-Veh and -Tx mice (Fig. 4b) indicates that loss of PKM2 accelerates CSC function but not growth rate. Moreover, we found that colon polyps of PKM2^ΔLgr5^-Tx mice contained more Lgr5^+^ cells, a marker for stem-like cells in CRC^31^, than those of Veh-treated mice (Fig. 4c). To elucidate whether PKM2 loss in cancer cells is linked to the increase of CSC-like function, we next isolated EpCAM^+^Lgr5^+^ cells from polyps and cultured single Lgr5^+^ CSC without Wnt3a and R-spondin. Lgr5^+^ CSC without PKM2 displayed higher organoid-forming capacity than CSC with PKM2 (Fig. 4d). While organoids obtained from colon polyps of PKM2^ΔLgr5^-Veh mice were all PKM2-positive, dominant expression of PKM1 was found in cancer organoids from colon polyps of PKM2^ΔLgr5^-Tx mice (Fig. 4e,f). Ki67-positive cells were comparable between PKM1-positive and -negative cancer organoids (Fig. 4e). These results suggest that PKM2 deletion in Lgr5^+^ ISC drove an increase in CSC-like function.

### Metabolites involved in energy metabolism are altered in cancer tissues and organoids of PKM2-deficient mice

**Figure 5:**
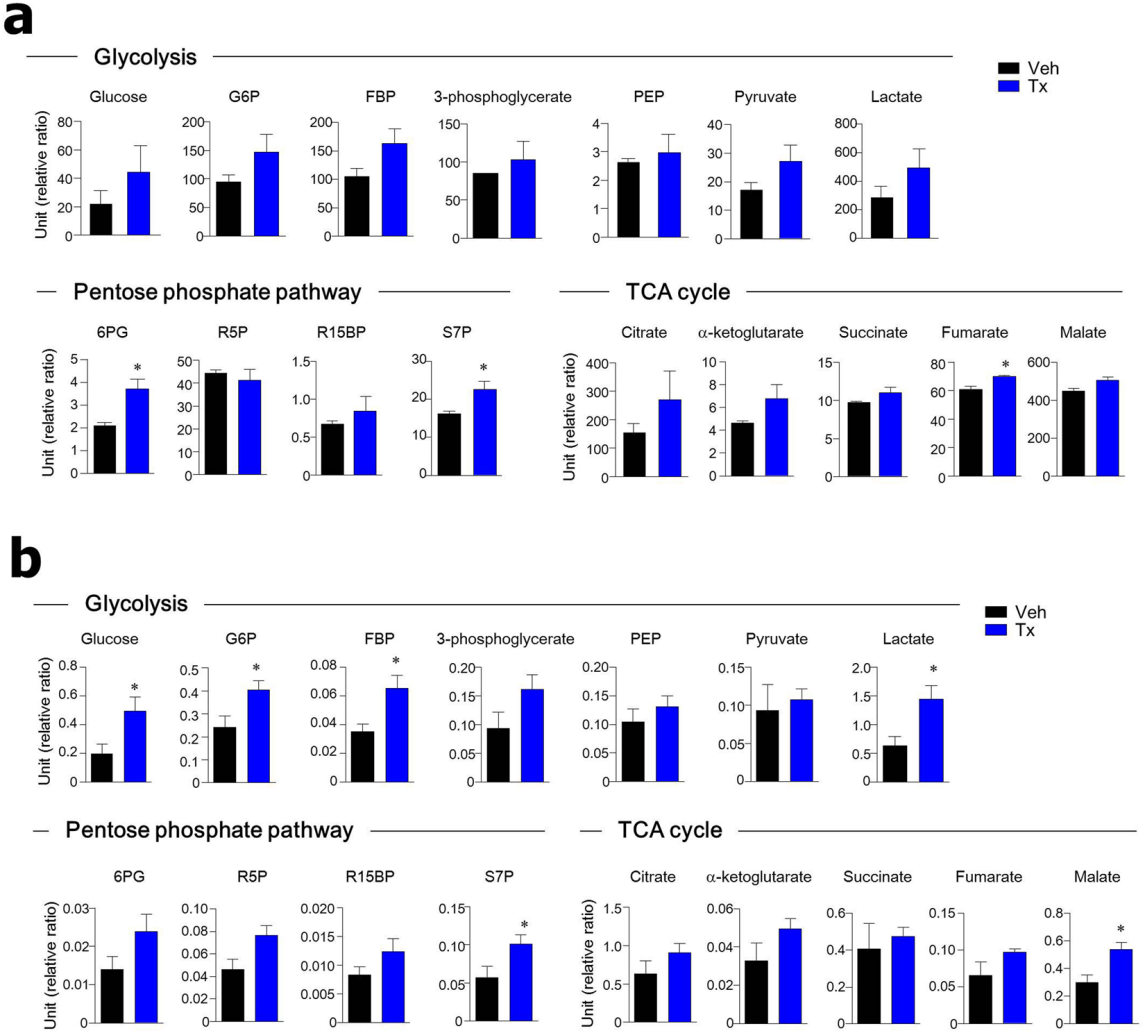
Metabolite levels in energy metabolism are altered in cancer tissues and organoids of PKM2^ΔLgr5^ mice. Relative concentration of metabolic intermediates in energy metabolism in colon tissues (n = 3) (**a**) and organoids from colon polyps (n = 4–6) (**b**) of AOM/DSS-treated PKM2^ΔLgr5^–Veh and –Tx mice. Metabolomic analysis was performed by liquid chromatography-tandem mass spectrometry. Relative ratio is peak area ratio of each analysis vs. internal standard. All data are mean ± s.e.m. Statistical analyses were done by Student’s *t*-test. *p < 0.05.

Since PKM2 is an essential enzyme for the metabolic reprogramming of cancer cells, we investigated the metabolites of colon polyps (Fig. 5a) and of organoids from colon polyps (Fig. 5b) in the absence of PKM2. While there were no significant changes within the glycolytic pathway, intermediates of the pentose phosphate pathway such as 6-phosphogluconate (6PG), sedoheptulose-7-phosphate (S7P), and tricarboxylic acid (TCA) cycle intermediates (e.g., fumarate) were higher in colon polyps of AOM/DSS-treated PKM2^ΔLgr5^-Tx mice than in PKM2^ΔLgr5^-Veh mice (Fig. 5a). We further found that in the glycolytic pathway, glucose, glucose-6-phosphate (G6P), fructose-1, 6-bisphosphate (FBP), and lactate were significantly higher in cancer organoids of PKM2^ΔLgr5^-Tx mice than in PKM2^ΔLgr5^-Veh mice. In addition, high levels of intermediates of the pentose phosphate pathway, such as S7P and the TCA cycle (e.g., malate), were found in cancer organoids from PKM2-deficient mice but not in PKM2-intact mice (Fig. 5b). Overall, PKM2-deficiency in Lgr5^+^ cells accelerated intermediates related to glycolytic, pentose phosphate, and TCA cycle pathways in colon polyps.

### PKM2 loss increases mitochondrial ATP production

**Figure 6:**
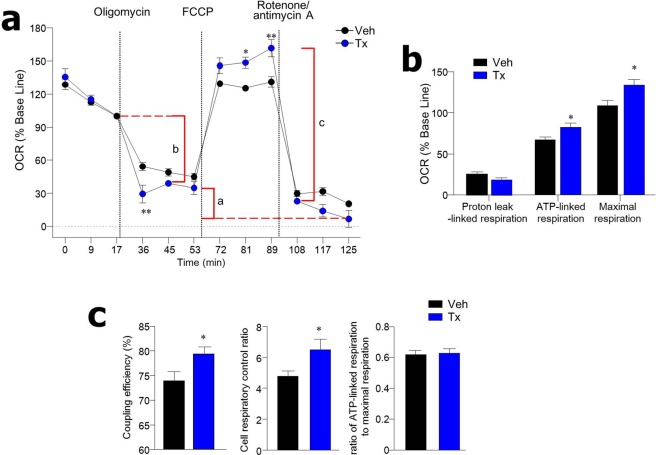
Organoids from colon polyps of tamoxifen (Tx)-treated PKM2^ΔLgr5^ mice have high rates of mitochondrial ATP production. (**a**) Oxygen consumption rate (OCR) of organoids from colon polyps of AOM/DSS-treated PKM2^ΔLgr5^–Veh and –Tx mice (n = 6/group). Results shown are percentage of baseline measurement for each treatment. (**b**) Individual parameters for proton leak-linked respiration, ATP-linked respiration and maximal respiration from panel A. Parameters were calculated with average value of three time points in each step. (**c**) Coupling efficiency (100 × ATP-linked respiration/basal respiration), cell respiratory control ratio (maximal respiration/proton leak), and ratio of ATP-linked respiration to maximal respiration (ATP-linked respiration/maximal respiration) were calculated from absolute values of OCR after normalization to protein levels. Parameters were calculated with average value of three time points in each step. Data are representative of two independent experiments. All data are mean ± s.e.m. Statistical analyses were done by Student’s *t*-test. *p < 0.05, **p < 0.01.

Since PKM2 contributes to the metabolic switch from mitochondrial oxidative phosphorylation (OXPHOS) to aerobic glycolysis in cancer cells^3^, we next addressed oxygen consumption rate (OCR), an indicator of mitochondrial respiration, in the absence of PKM2. After treatment with the ATP synthase inhibitor oligomycin, average OCR of cancer organoids from colon polyps of PKM2^ΔLgr5^-Tx mice was lower than in those from PKM2^ΔLgr5^-Veh mice (Fig. 6a). Of note, addition of the uncoupling agent carbonyl cyanide-4-(trifluoromethoxy) phenylhydrazone (FCCP) led to a considerable increase in maximal respiration of cancer organoids from colon polyps of PKM2^ΔLgr5^-Tx mice when compared to those of PKM2^ΔLgr5^-Veh mice (Fig. 6a,b). ATP-linked respiration and coupling efficiency [100 × (ATP-linked respiration/basal respiration)], both defined as the respiration that is used to drive mitochondrial ATP synthesis, was higher in cancer organoids from colon polyps of PKM2^ΔLgr5^-Tx mice than in PKM2^ΔLgr5^-Veh mice (Fig. 6b,c). In addition, the cell respiratory control ratio (maximal respiration/proton leak), the general indicator for mitochondrial function, was higher in cancer organoids from PKM2^ΔLgr5^-Tx mice than from PKM2^ΔLgr5^-Veh mice (Fig. 6c). Colon crypts of naïve PKM2^ΔLgr5^-Tx mice also exhibited increased maximal respiration and cell respiratory control ratio, suggesting that PKM2 loss led to enhanced mitochondrial oxidation capacity (Fig. S4). These results indicate that PKM2 deficiency in ISC results in activation of mitochondrial ATP production in colon polyps of CRC-induced mice.

### Genes associated with activation of the Wnt pathway and tumor progression are increased in cancer tissues and organoids of PKM2-deficient mice

**Figure 7:**
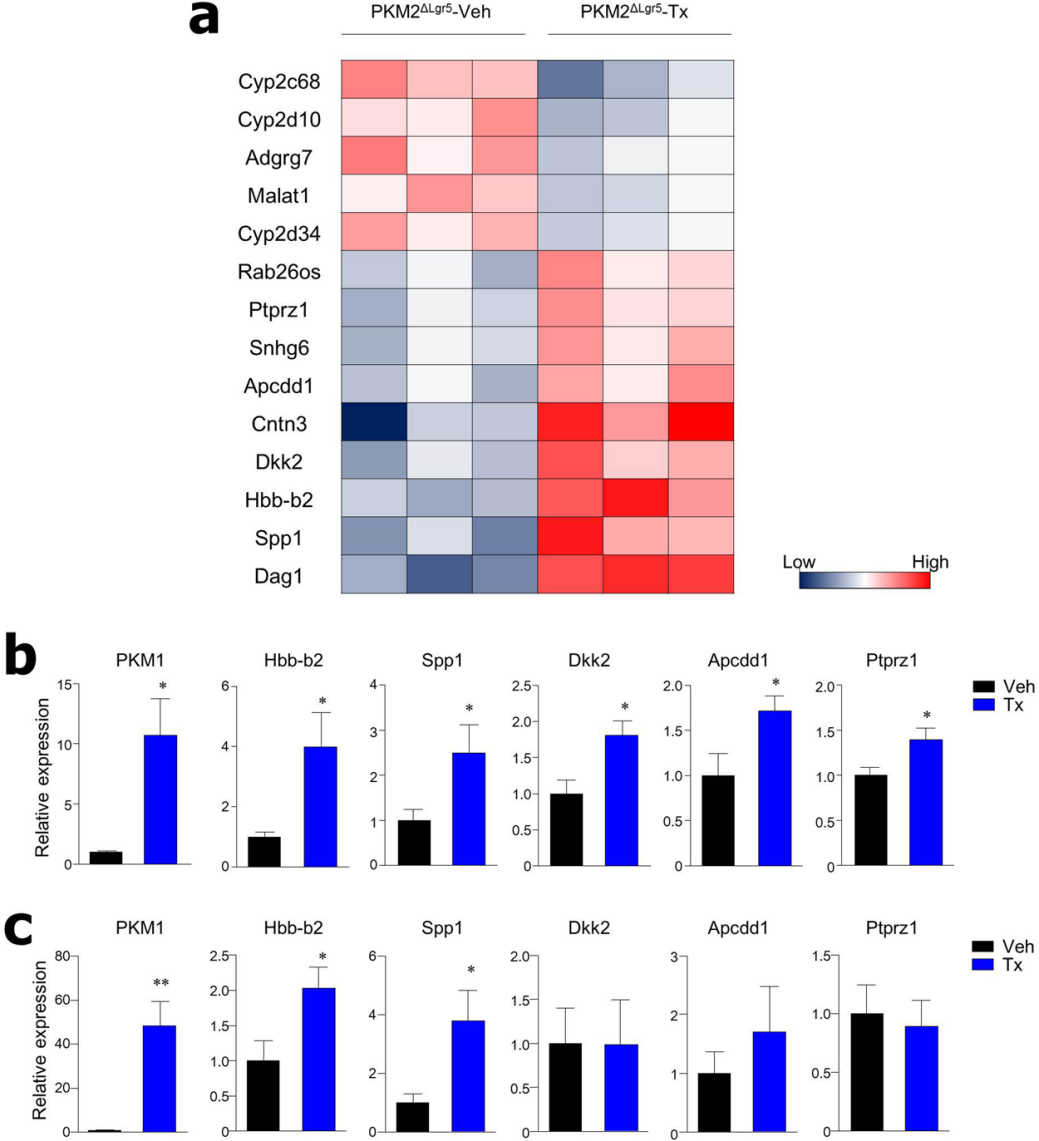
Genes associated with activation of Wnt pathway and tumor progression are increased in colon tissues and colon polyp organoids from PKM2^ΔLgr5^ mice. (**a**) RNA-seq analysis of colon tissues from AOM/DSS-treated PKM2^ΔLgr5^–Veh and –Tx mice (n = 3/group). Differentially expressed genes (fold change > 1.5 and *p* < 0.05) were selected and clustered in a heat map. Relative mRNA levels of selected genes were measured by real-time PCR in colon tissues (n = 5/group) (**b**) and organoids from colon polyps (n = 8/group) (**c**) from AOM/DSS-treated PKM2^ΔLgr5^ mice. *p < 0.05, **p < 0.01.

We performed RNA-sequencing (RNA-seq) experiments using colon polyps from PKM2^ΔLgr5^-Tx and -Veh mice after treatment with AOM/DSS. Among 37 genes significantly differentially expressed between the two groups, 19 genes showed significant up-regulation (fold-change, ≥1.5; *p* < 0.05) in colon polyps from PKM2^ΔLgr5^-Tx when compared with PKM2^ΔLgr5^-Veh mice (Fig. 7a). *Spp1*^32^, *Dkk2*^33^, and *Apcdd1*^34^, Wnt target genes and well-known genes that enhance tumor growth and metastasis, were highly expressed in colon polyps from PKM2^ΔLgr5^-Tx mice but not from PKM2^ΔLgr5^-Veh mice. In contrast, 18 genes were down-regulated in colon polyps from PKM2^ΔLgr5^-Tx mice including cytochrome P450 (CYP) 2 family (Fig. 7a). Gene expression patterns were further addressed in colon polyp tissues (Fig. 7b) and cancer organoids from colon polyps (Fig. 7c) by real-time PCR. Of note, mRNA levels of *PKM1*, *Hbb-b2*, and *Spp1* were significantly elevated in both colon polyp tissues and cancer organoids from colon polyps from PKM2^ΔLgr5^-Tx mice. Taken together, these results further support the conclusion that PKM2-deficiency in ISC accelerates progression of CRC in the murine model.

## Discussion

In this study, we demonstrated that despite high PKM2 expression in patients with UC or CRC and its correlation with poor clinical outcomes in patient-derived genomic data, development of inflammation-induced CRC was accelerated in mice with the deletion of PKM2 in Lgr5^+^ ISC or in intestinal epithelial cells. We similarly observed enhanced formation of cancer organoids obtained from colon polyps of PKM2^ΔLgr5^ -Tx mice after AOM/DSS treatment. Of note, specific deletion of PKM2 activated PKM1 expression in colon tissues and organoids in steady-state and CRC conditions. In addition, genes associated with tumor growth and metastasis were elevated in CRC-induced mice lacking PKM2 in ISC. Furthermore, the deficiency of PKM2 contributed to increased mitochondrial oxygen consumption and the shift of metabolites involved in energy metabolism. These results indicate that loss of PKM2 in an ISC-specific manner promotes development of inflammation-induced CRC. Our observation and those of others^10,35^ of accelerated tumor progression after PKM2 deletion suggests that the blockade of PKM2 for cancer therapy might not be an effective approach.

PKM2 expression has been considered a hallmark of cancer; however, recent studies have yielded contradictory results regarding the requirement for PKM2 in tumor growth. Mice deficient in PKM2 have showed enhanced tumorigenesis in several experimental models^10,35,36^. In our current study, inflammation-induced CRC was more severe when there was PKM2 loss in ISC, suggesting that PKM2 is not absolutely required for tumor maintenance and growth in the colon. Similar results were observed in an APC-driven colon cancer model^37^. We found significant levels of PKM1 were activated when PKM2 was deleted in both colon tissues and organoids from colon polyps (Figs 3 and 4). It has been reported that the expression of PKM1, which resulted from PKM2 loss, was found only in non-proliferating cells in breast cancer^10^. However, we observed that proliferation of PKM1-expressed cancer cells was similar to that of cells expressing PKM2 in inflammation-induced CRC (Fig. 3c), consistent with other studies^11,38^. The most recent study revealed that PKM1 promotes tumor growth in mouse lines expressing PKM1 or PKM2^38^. PKM1-activated glucose catabolism and PKM1-depedent autophagy contributed to malignancy (e.g., small-cell lung cancer). Although we need to explore this further, we speculate that compensatory expression of PKM1 by deletion of PKM2 provides metabolic advantage to support the expansion of cancer cells in different ways than PKM2.

We observed increased formation of organoids in PKM2-deficient colon polyps in the absence of Wnt activators (i.e., Wnt3a and R-spondin1) (Fig. 4). As reported previously, the majority of adenoma organoids proliferate and propagate in the absence of exogenous Wnt activators^39^. The fact that Wnt activators-independent CRC organoids had mutations in the Wnt signaling pathway (e.g., *Apc*, *Ctnnb1*, and *Tcf7l2*)^39^, suggests that cancer cells from PKM2-deficient mice might carry those mutations. However, we could not find changes of oncogenes such as *Apc*, *Ctnnb1*, and *Tcf7l2*; instead we found up-regulated levels of genes such as *Spp1*, *Dkk2*, and *Apcdd1*, which are associated with aberrant Wnt/β-catenin signaling (Fig. 7). For instance, osteopontin, encoded by *Spp1*, led to β-catenin stabilization and nuclear translocation via *Akt*-mediated GSK-3β inhibition^40^. Moreover, overexpression of osteopontin was detected in mice with activation of the Wnt pathway via mutation of *Apc*^41^. One recent study demonstrated elevation of *Dkk2* expression in CRC of mice with *Apc* mutation and found that treatment of small interfering RNAs for β-catenin suppressed the *Dkk2* upregulation^42^. Another study showed that *Apcdd1* is a direct target gene of the β-catenin/Tcf complex^34^. A potential mechanism suggested by others is that PKM2 negatively regulates β-catenin via miR-200a, inhibiting β-catenin translation^43^. When taken together, our findings imply that PKM2 deficiency enhances Wnt signaling pathway-related genes and accelerates the tumorigenic process of inflammation-induced CRC.

Our results indicate an increase in the forming efficiency of cancer organoids from PKM2-deficient mice after passage of cancer organoids (Fig. 4b). In addition, the population of Lgr5^+^ CSC was elevated in cancer tissues from PKM2^ΔLgr5^-Tx mice (Fig. 4c). Single Lgr5^+^ CSC with PKM2 depletion formed organoids at a higher frequency than CSC with PKM2 (Fig. 4d). We speculate that CSC-like cells are enriched in cancer tissues of mice with PKM2 loss. Moreover, cancer organoids from PKM2^ΔLgr5^-Tx mice showed an elevation of ATP-linked respiration and mitochondrial function (Fig. 6), which might result from compensatory expression of PKM1. Although there is no consensus on whether CSC rely on aerobic glycolysis or OXPHOS, there is increasing evidence that CSC adopt mitochondrial oxidative metabolism^44,45^. Colon CSC have increased mitochondrial function, and their stemness is regulated by the maintenance of mitochondrial function^46^. Previous studies reported that cancer cell lines resistant to anticancer drugs (i.e., fluorouracil and oxaliplatin) exhibited an up-regulation of PKM1 expression, OXPHOS, and stem-like traits including the expression of CD133 and formation of anchorage-independent spheres^47,48^. Considering these findings, we speculate that increased self-renewal of CSC-like cells in cancer tissues from PKM2^ΔLgr5^-Tx mice might be due to PKM1-induced shifts of cancer metabolism.

Although cancer tissues from PKM2-deficient and -intact mice contained nearly equal amounts of glycolytic intermediates, several metabolites in glycolysis were elevated in cancer organoids from colon polyps with PKM2 loss (Fig. 5). This discrepancy might be related to the fact that cancer organoids were in relatively higher glucose culture conditions than the cancer tissues, which were in low-glucose tumor microenvironment conditions. A recent study revealed that cancer cells expressing only PKM1 increased the flux of glucose through glycolysis and the TCA cycle^38^. We speculate that PKM1 expression in PKM2-deficient cancer organoids contributes to the increase of glycolysis in a glucose-sufficient condition and to entry of glucose into the TCA cycle. In addition, cancer tissues elevate some intermediates in the pentose phosphate pathway. In another recent study, downregulated PKM2 resulted in an increase of the NADPH/NADP ratio in pancreatic cancer cells during hypo-glucose conditions, suggestive of activation of the pentose phosphate pathway^49^. When all of these findings are taken together, it appears that depletion of PKM2 might be involved in a shift of glucose flux to the pentose phosphate pathway under hypo-glucose conditions.

Overall, our study demonstrates that loss of PKM2 accelerates the progression of colitis-induced CRC by AOM/DSS treatment. We found the enhanced Wnt/β-catenin pathway and CSC-like function in cancer organoids in the absence of PKM2. Recent studies on targeting PKM2 function in cancer cells showed that the depletion of PKM2 drove the activation of other compensatory pathways for survival. These compensatory functions may promote the development of cancer. These findings suggest that therapeutic strategies targeting cancer metabolism require caution and further study.

## Materials and Methods

### Ethics statement

All animal experiments were approved by the Institutional Animal Care and Use Committee of Asan Medical Center (Seoul, Korea) (Approval No. 2016-12-131). Animal experiments were performed under anesthesia with a mixture of ketamine (100 mg/kg) and xylazine (20 mg/kg), and all efforts were made to minimize suffering. All experiments were performed in accordance with relevant guidelines and regulations.

### Mice

PKM2^f/f^, Lgr5-EGFP-IRES-creERT2, and Villin-cre mice were purchased from Jackson Laboratory (Bar Harbor, ME). All mice were maintained under specific pathogen-free conditions in the animal facility at Asan Medical Center, where they received sterilized food and water *ad libitum*. For Lgr5^+^ cell-specific deletion of PKM2, PKM2^f/f^xLgr5^CreERT2^ mice at age 6–8 weeks were injected intraperitoneally with 1 mg of tamoxifen (MP Biomedicals, Aurora, OH) in sunflower seed oil (Sigma Aldrich, St. Louis, MO) once a day for 5 consecutive days. PKM2^f/f^xLgr5^CreERT2^ mice were injected with sunflower seed oil alone (vehicle) for control.

### Experimental colitis-associated colorectal cancer

Male mice were given a single intraperitoneal injection of AOM (Sigma Aldrich) (12.5 mg/kg body weight) in combination with three cycles of DSS (molecular weight 36,000–50,000; MP Biomedicals) treatment as illustrated in Fig. 2a. Body weight was monitored twice a week.

### Histological scoring

The entire colon was removed, opened longitudinally, and scored for polyp numbers. Colon tissues were then formed into Swiss rolls, fixed in 4% paraformaldehyde (PFA), and embedded in paraffin. Tissue sections were stained with hematoxylin-eosin (H&E). Histological scoring was performed blindly by pathologists. Tumors were graded and scored as 1 = low-grade dysplasia, 2 = high-grade dysplasia, 3 = intramucosal adenocarcinoma, 4 = invasive adenocarcinoma. Area affected by dysplasia was scored as 1, <10%; 2, 10–25%; 3, 25–50%; 4, >50% of colon.

### Culture of organoid using colon polyps and sorted Lgr5^+^ cells

Colon polyps were removed and washed with PBS containing gentamicin (50 μg/ml, Thermo Fisher, Waltham, MA). Tissues were minced with a scissor and incubated for 1 h in RPMI 1640 medium (Thermo Fisher) containing collagenase IV (2.5 mg/ml, Sigma Aldrich), DNase (0.2 mg/ml, Sigma Aldrich) at 37 °C with stirring. Cell suspensions were filtered and centrifuged at 500 × g for 5 min. For construction of organoids, the dissociated cells were seeded at concentrations of 2 × 10^4^ cells per well with Matrigel (Corning, Corning, NY) in a 24-well plate. For isolation of Lgr5-GFP^+^ cells, cell pellets were incubated with purified anti- mouse CD16/32 antibody (BD Biosciences, Franklin Lakes, NJ), followed by staining with Live/Dead Cell Stain kit (Thermo Fisher) and EpCAM (clone G8.8, Thermo Fisher). Cell sorting was performed using a FACS AriaIII cell sorter (BD Biosciences). Sorted cells were seeded at 1 × 10^4^ cells per well with Matrigel. All organoids were cultured in EN medium containing B27 supplement (Thermo Fisher), N2 supplement (Thermo Fisher), EGF (R&D Systems, Minneapolis, MN), Noggin (R&D Systems), N-acetyl cysteine (Sigma Aldrich), and penicillin-streptomycin (Thermo Fisher) in advanced DMEM/F12 (Thermo Fisher). EN medium was replaced every 2–3 days. For subculture, dissociation of organoids was carried out by resuspending organoids in TrypLE Express (Thermo Fisher) for 10 min at 37 °C.

### Immunofluorescence staining

Colon tissues were fixed with 4% PFA and dehydrated with 15% and 30% sucrose in PBS. Dehydrated tissues were then embedded in frozen section compound, frozen, and sliced into 6-μm sections. Tissue sections were fixed with −20 °C acetone for 5 min, blocked with PBS containing 5% BSA for 1 h at room temperature (RT), and stained with primary antibodies overnight at 4 °C. Tissues were washed in PBS, incubated with secondary antibodies at RT for 1 h, stained with 4′,6-diamidino-2-phenylindole (DAPI; Thermo Fisher) for 2 min at RT, and mounted with PermaFluor mountant (Thermo Fisher). For staining, organoids seeded in an eight-well chamber (Thermo Fisher) were fixed with 4% PFA in PBS for 10 min at RT. After organoids were washed in PBS and subsequently permeabilized in PBS containing 0.5% Triton X-100 for 20 min at RT, they were blocked with 0.5% BSA in PBS for 1 h at RT. After staining, images were captured on an LSM 710 confocal microscope (Carl Zeiss, Oberkochen, Germany). Primary antibodies were rabbit anti-PKM1 (clone D30G6), rabbit anti-PKM2 (clone D78A4), rat anti-Ki67 (clone 11F6), and mouse anti-β-catenin (clone 14). Secondary antibodies were Alexa Fluor 546 donkey anti-rabbit IgG, Alexa Fluor 647 donkey anti-rabbit IgG, Alexa Fluor 488 goat anti-mouse IgG, Alexa fluor 594 goat anti-rat IgG. Antibodies for analysis were from Cell Signaling Technology (Danvers, MA), BD Biosciences, Abcam (Cambridge, UK), and BioLegend (San Diego, CA).

### Metabolomics

Colon polyp tissues (22 ± 1 mg) were homogenized using a TissueLyzer (Qiagen, Valencia, CA) with chloroform/methanol (2/1, v/v). The homogenate was incubated at 4 °C for 20 min. The internal standard (^13^C_5_ Glutamine-d_4_) was added to the sample, followed by centrifugation. After collection of supernatant, H_2_O was added and centrifuged. For metabolomics analysis, organoids were washed and lysed using cold methanol/H_2_O (80/20, v/v) by vigorous vortexing, and then centrifuged. Aqueous phase samples were dried by vacuum centrifuge and reconstituted with 50% methanol. Metabolites were analyzed with an liquid chromatography-tandem mass spectrometry (LC-MS/MS) system equipped with a 1290 high-performance liquid chromatography system (Agilent Technologies, Santa Clara, CA), Qtrap 5500 (AB Sciex, Framingham, MA), and reverse-phase column (Synergi fusion RP 50 × 2 mm; Phenomenex, Torrance, CA). Multiple reaction monitoring was used in negative-ion mode and the extracted ion chromatogram (EIC) corresponding to the specific transition for each metabolite was used for quantitation. The area under the curve of the EIC was normalized to that of the EIC of the internal standard. The peak area ratio of each metabolite to internal standard was normalized using the protein concentration or the weight of each sample.

### Measurement of OCR by XF24 Flux Analyzer

Organoids were dissociated with TrypLE Express for 10 min at 37 °C. Dissociated single cells were seeded at 5 × 10^3^ cells per well with Matrigel in XF24 cell culture microplates (Agilent Technologies) and cultured in EN medium for 5–6 days. One hour before measurement, culture medium was replaced with OCR assay media [minimal DMEM (Sigma Aldrich) supplemented with GlutaMAX (2 mM, Thermo Fisher), pyruvate (5 mM, Thermo Fisher), glucose (20 mM, Junsei Chemical, Tokyo, Japan)] in the 37 °C non-CO_2_ incubator for 1 h. We used a Seahorse Bioscience XF24 analyzer (Agilent Technologies) to measure OCR. Oligomycin (1 μM), FCCP (1 μM), and rotenone and antimycin (1 μM) were injected for OCR measurements. All reagents were purchased from Sigma Aldrich. After the measurements, cells were lysed with RIPA buffer (Thermo Fisher) and isolated proteins were quantified with Pierce BCA Protein Assay Kit (Thermo Fisher) for normalization.

### RNA-seq analysis

RNA from colon polyps of AOM/DSS-treated mice was isolated using *mir*Vana miRNA isolation kit (Thermo Fisher). A library was prepared with 1 μg of total RNA for each sample by TruSeq mRNA Sample Prep kit (Illumina, San Diego, CA). The protocol consisted of polyA-selected RNA extraction, RNA fragmentation, random hexamer-primed reverse transcription, and 100 nt paired-end sequencing by the HiSeq4000 platform (Illumina). The libraries were quantified using qPCR according to the qPCR Quantification Protocol Guide (KAPA Library Quantification kits for Illumina Sequencing platforms) and qualified using the 2100 Bioanalyzer (Agilent Technologies). RNA-seq experiments and statistical analysis were performed by Macrogen, Inc. (Seoul, Korea).

### Western blot

The colonic epithelial cells from naïve mice or colon polyps from AOM/DSS-treated mice were lysed in RIPA buffer (Thermo Fisher) with protease inhibitor (Sigma Aldrich). Supernatants were collected by centrifuge at 11,000 × g for 10 min. Concentrations of proteins in the supernatant were determined using Pierce^TM^ BCA Protein Assay Kit (Thermo Fisher). Proteins were boiled with Laemmli sample buffer and separated with 10% SDS-PAGE. Proteins were blotted onto a PVDF membrane (Merck Millipore, Burlington, MA). After membranes were blocked with 5% skim milk in TBST for 1 h, they were incubated overnight at 4 °C with the respective primary antibodies against PKM1, PKM2, PKM (clone C103A3, Cell Signaling Technology) or β-actin (Cell Signaling Technology), diluted in blocking buffer. Membranes were washed with TBST buffer and incubated with an appropriate HRP-conjugated secondary antibody (Cell Signaling Technology) for 2 h at RT. Signals were developed with enhanced chemiluminescence (DoGEN, Seoul, Korea) and visualized using ImageQuant LAS 4000 (GE Healthcare, Buckinghamshire, UK). Relative band intensity was normalized to β-actin and quantified by ImageJ 1.48 open source software (https://imagej.nih.gov/ij/).

### Statistics

GraphPad Prism software (GraphPad, La Jolla, CA) was used for statistical analysis. Significant differences between two groups were analyzed with two-tailed unpaired *t*-test. Multiple groups were analyzed by one- or two-way ANOVA followed by Bonferroni’s *post hoc* test (*p < 0.05; **p < 0.01).

## Supplementary information

Dataset 1
