# Supplementary material for: Loss of PKM2 in Lgr5+ intestinal stem cells promotes colitis-associated colorectal cancer
Source: Sci Rep. 2019 Apr 17;9:6212. doi: 10.1038/s41598-019-42707-8 (PMC6470145; doi:10.1038/s41598-019-42707-8)
Supplement: Supplementary file 1 — Dataset 1 [file 41598_2019_42707_MOESM1_ESM.pdf]

Loss of PKM2 in Lgr5^{+} intestinal stem cells promotes colitis-associated colorectal cancer

Yeji Kim[1], Yong-Soo Lee[1], Sung Wan Kang[1], Seungil Kim[1], Tae-Young Kim[1], Su-Hyun Lee[1], Sung Wook Hwang[1,2], Jihun Kim[3], Eun Na Kim[3], Jin-Sung Ju[4], Yun-Yong Park[4*], and Mi-Na Kweon[1*]

[1]*Mucosal Immunology Laboratory,* [4]*Department of Convergence Medicine, University of Ulsan College of Medicine / Asan Medical Center, Seoul, Republic of Korea.*

[2]*Department of Gastroenterology,* [3]*Department of Pathology, University of Ulsan College of Medicine / Asan Medical Center, Seoul, Republic of Korea*

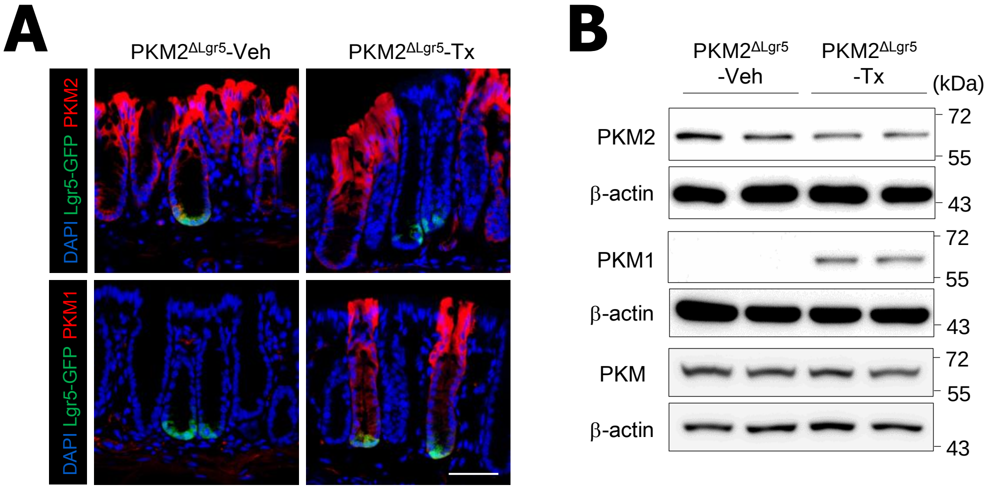

Figure S1. Deletion of PKM2 in Lgr5⁺ stem cells results in PKM1 expression in progeny cells. (**A**) Immunofluorescence images of PKM2 and PKM1 expression and (**B**) western blot analysis of colonic epithelial cells in steady-state from PKM2$^{\Delta Lgr5}$–Veh and –Tx mice. The blots were cropped. The full-length blots are presented in Supplementary Fig. S5A.

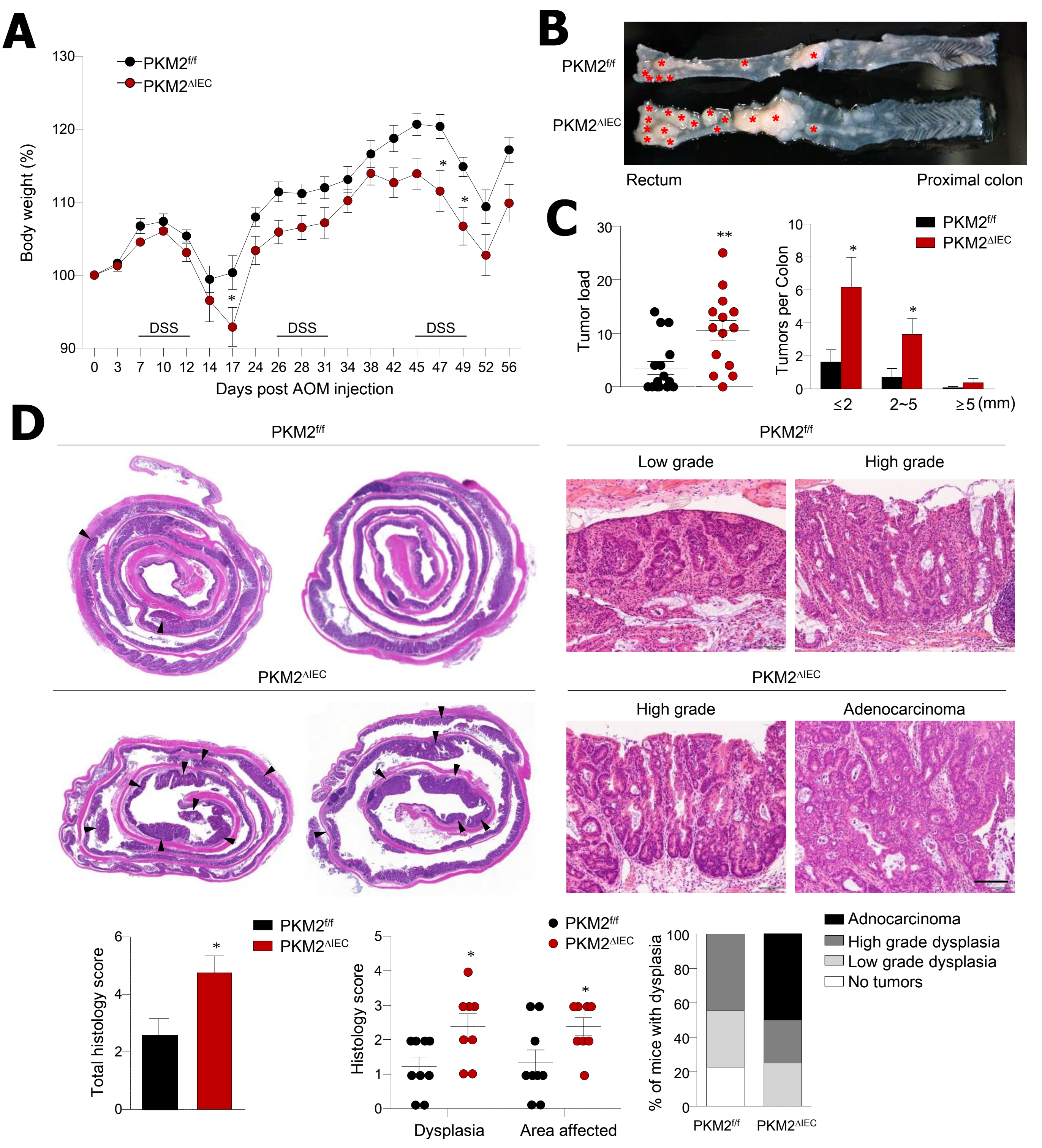

Figure S2. PKM2 deletion in intestinal epithelial cells promotes formation of colitis-associated colon cancer. (**A**) Weight loss (n = 17) and (**B**) representative images of colons from AOM/DSS-treated PKM2$^{f/f}$ and PKM2$^{f/f}$ x Villin^{Cre} (PKM2$^{\Delta IEC}$) mice. (**C**) Tumor load (n = 16 for PKM2$^{f/f}$, n = 14 for PKM2$^{\Delta IEC}$) and polyp size (n = 14) in AOM/DSS-treated mice. (**D**) Colon histology of AOM/DSS-treated mice. Arrowheads indicate colon polyps. Representative images of low- and high-grade dysplasia and adenocarcinoma. Scale bar = 100 μm. H&E stained sections were scored for severity and area of dysplasia (n = 9 for PKM2$^{f/f}$, n = 8 for PKM2$^{\Delta IEC}$). All data are mean ± s.e.m. Statistical analyses were done by Student's *t*-test or two-way ANOVA with Bonferroni *post-hoc* test. *$p < 0.05$, **$p < 0.01$.

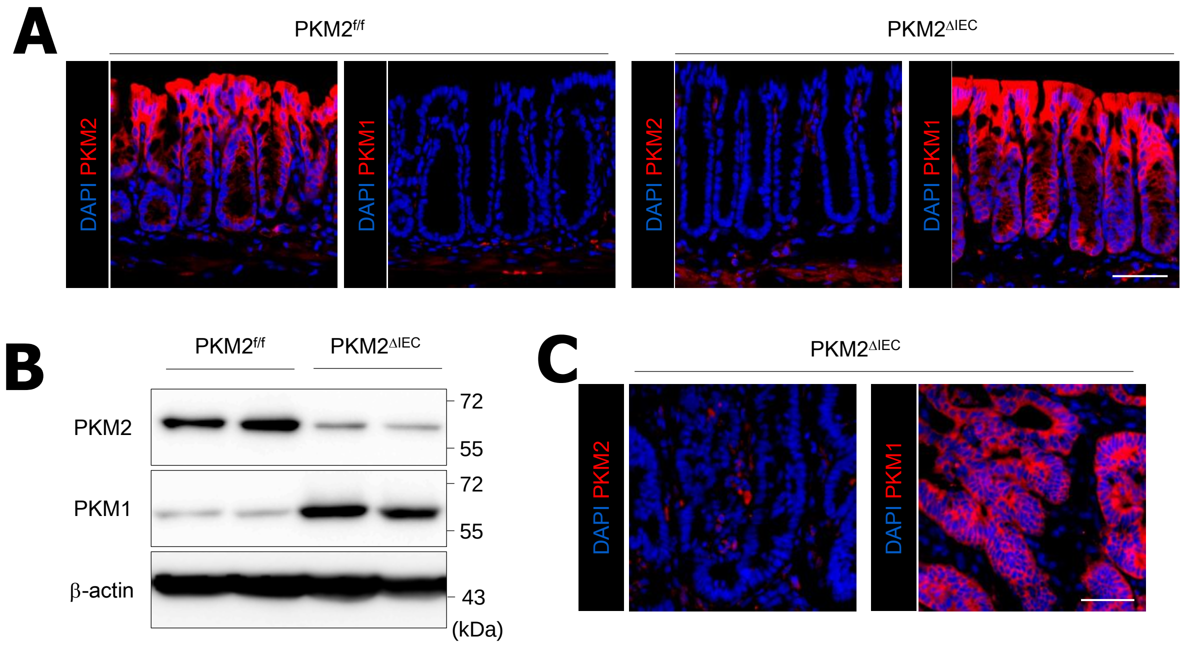

Figure S3. Deletion of PKM2 in intestinal epithelial cells drives PKM1 expression. (**A**) Immunofluorescence image and (**B**) western blot analysis of PKM2 and PKM1 expression in colons of PKM2$^{f/f}$ and PKM2$^{\Delta IEC}$ mice. The blots were cropped. PKM1, PKM2 and the control gene were run on the different gels. The full-length blots are presented in Supplementary Fig. S7. (**C**) Confocal analysis of PKM2 and PKM1 expression in colon polyps from AOM/DSS-treated PKM2$^{\Delta IEC}$ mice.

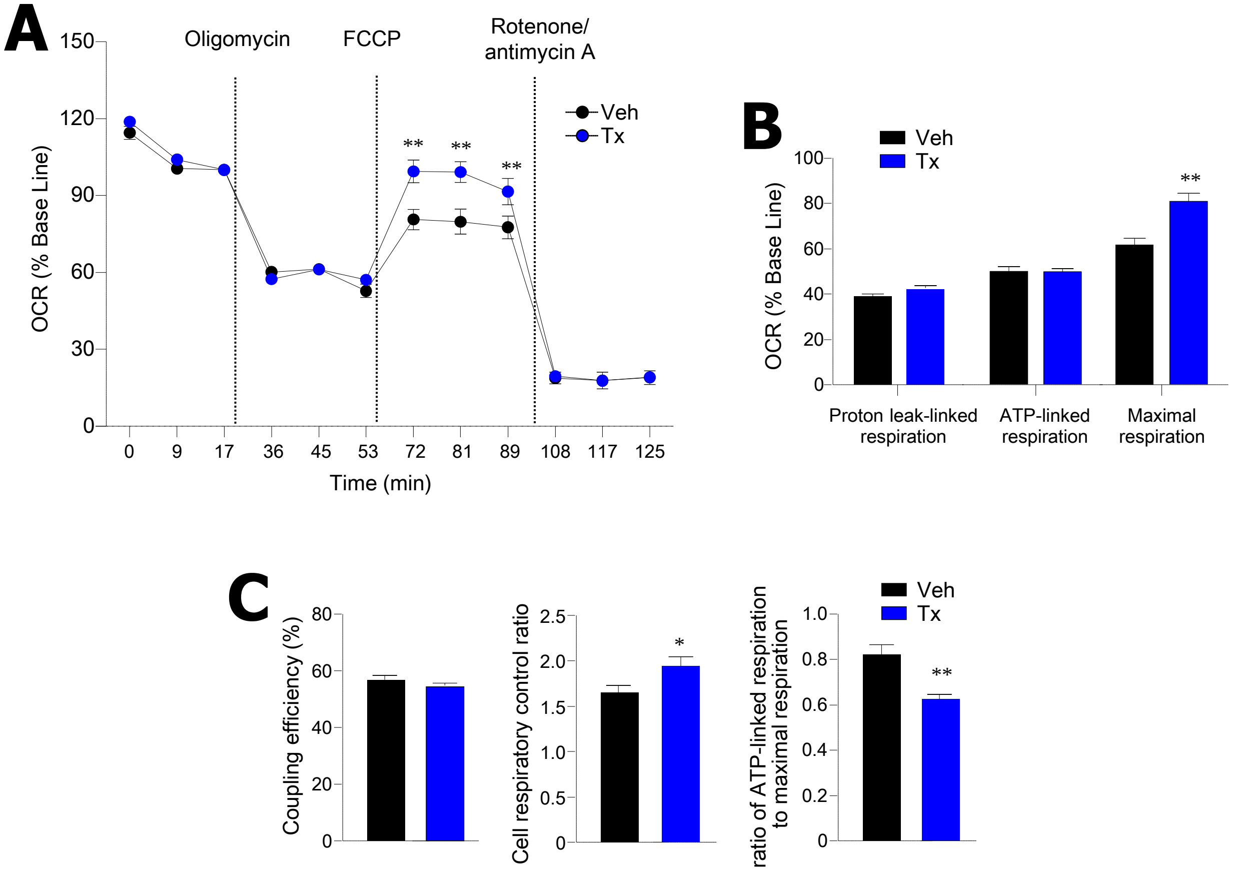

Figure S4. Deletion of PKM2 in Lgr5$^+$ stem cells alters mitochondrial function of colonic epithelial cells. (**A**) Oxygen consumption (OCR) of colonic epithelial cells from naïve PKM2$^{\Delta Lgr5}$–Veh and –Tx mice (n = 6/group). Results are shown as percentage of baseline measurement for each treatment. (**B**) Individual parameters for proton leak-linked respiration, ATP-linked respiration, and maximal respiration. Parameters were calculated with average values of three time points in each step. (**C**) Coupling efficiency (100 × ATP-linked respiration/basal respiration), cell respiratory control ratio (maximal respiration/proton leak), and ratio of ATP-linked respiration to maximal respiration (ATP-linked respiration/maximal respiration) were calculated from absolute values of OCR after normalization to protein levels. Parameters were calculated with average value of three time points in each step. Data are representative of two independent experiments. All data are mean ± s.e.m. Statistical analyses were done by Student's *t*-test. *$p < 0.05$, **$p < 0.01$.

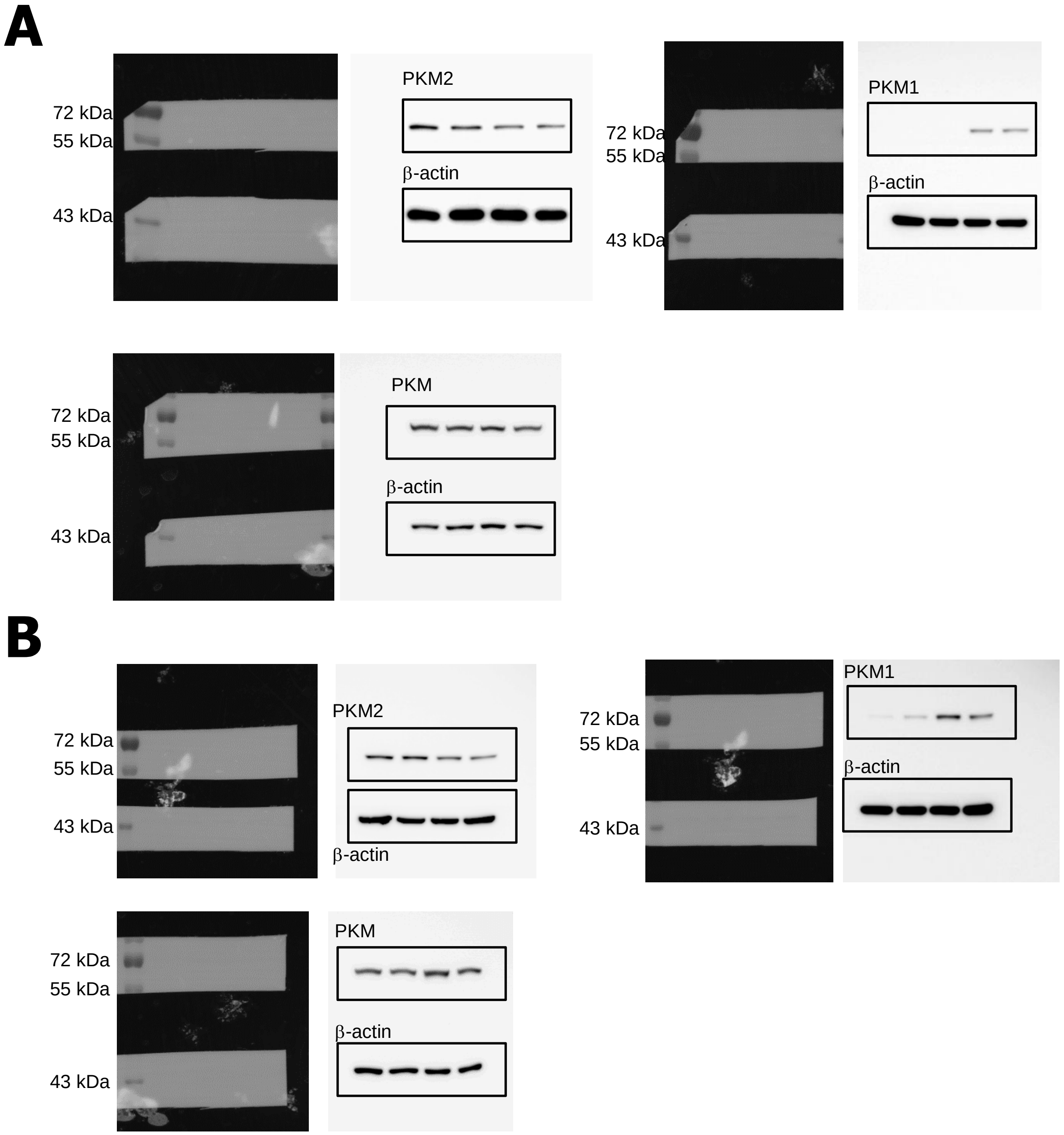

Figure S5. Original blots from Fig S1 and 3. (**A**) Blots of colonic epithelial cells in steady-state from PKM2$^{\Delta Lgr5}$–Veh and –Tx mice. (**B**) Blots of colon polyp tissues from AOM/DSS-treated PKM2$^{\Delta Lgr5}$ mice. Black lines show the cropping locations.

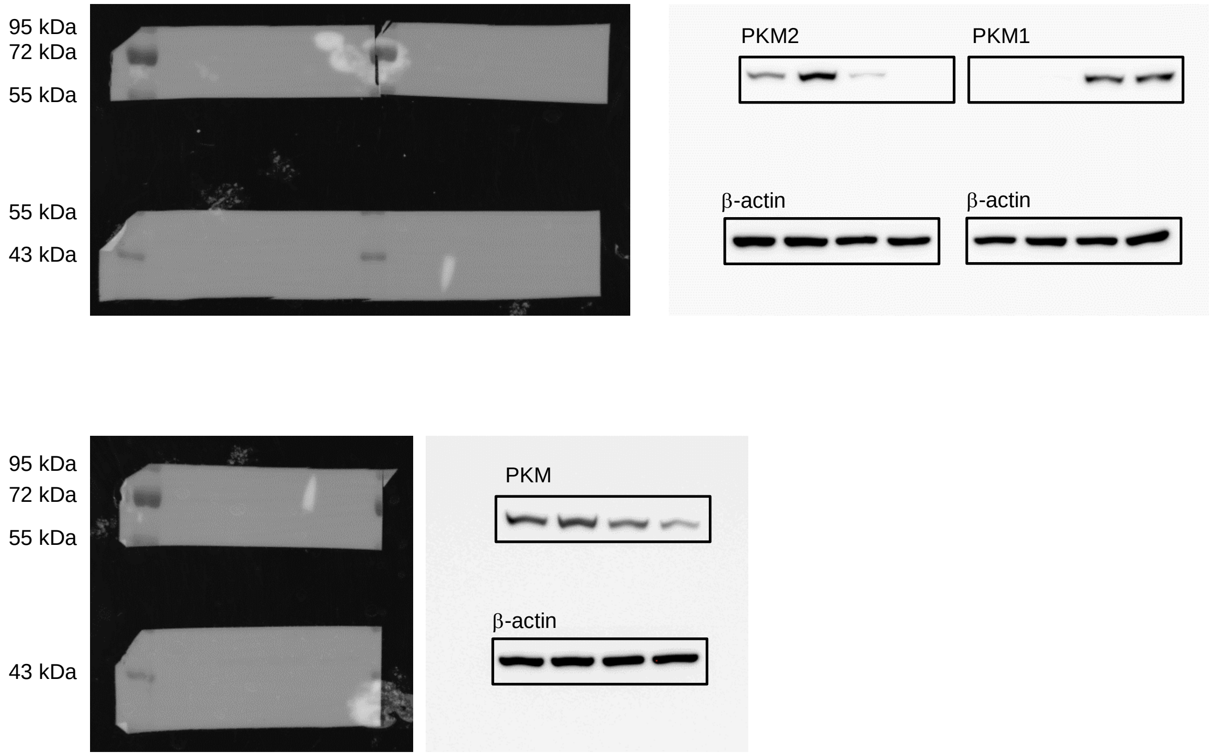

Figure S6. Original blots from Fig 4. Blots of cancer organoids isolated from colon polyps of $PKM2^{\Delta Lgr5}$–Veh and –Tx mice. Black lines show the cropping locations.

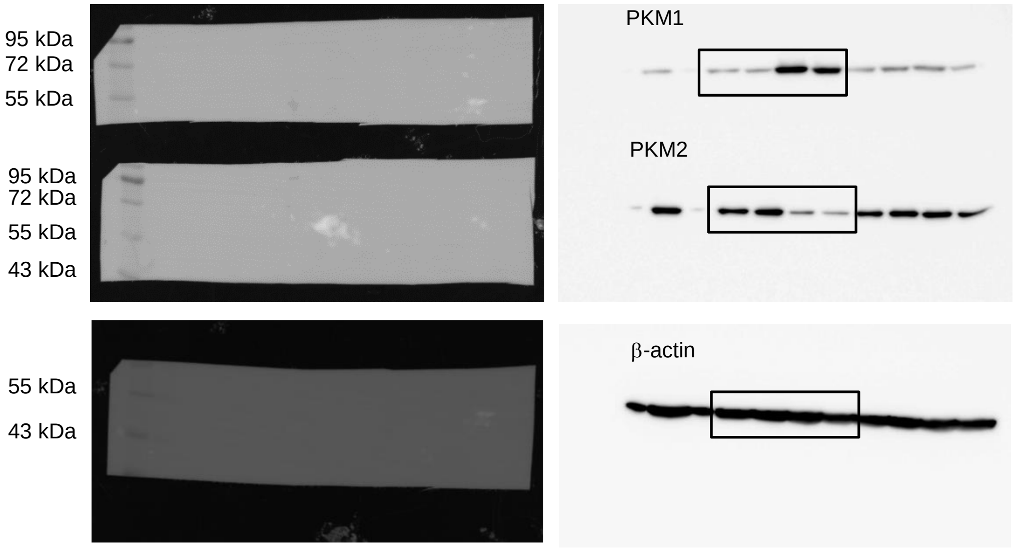

Figure S7. Original blots from Fig S3. Blots of colons from PKM2$^{f/f}$ and PKM2$^{\Delta IEC}$ mice. Black lines show the cropping locations.
